# Cryopreserved Spontaneous Spheroids from Compact Bone-Derived Mesenchymal Stromal Cells for Bone Tissue Engineering

**DOI:** 10.1089/ten.tec.2021.0001

**Published:** 2021-04-19

**Authors:** Hongwei Dong, Xianqi Li, Kai Chen, Ni Li, Hideaki Kagami

**Affiliations:** ^1^ Department of Hard Tissue Research, Graduate School of Oral Medicine, Matsumoto Dental University, Shiojiri, Japan.; ^2^ Department of Oral and Maxillofacial Surgery, School of Dentistry, Matsumoto Dental University, Shiojiri, Japan.; ^3^ Institute for Oral Science, Matsumoto Dental University, Shiojiri, Japan.; ^4^ Department of Stomatology, Shanghai Tenth People's Hospital, Tongji University School of Medicine, Shanghai, China.; ^5^ Department of General Medicine, IMSUT Hospital, The Institute of Medical Science, The University of Tokyo, Tokyo, Japan.

**Keywords:** cryopreservation, spontaneous spheroid, osteogenic differentiation capability, compact bone-derived mesenchymal stromal cells, MSCs

## Abstract

Spontaneously formed spheroids from mouse compact bone-derived mesenchymal stromal cells (CB-MSCs) possess enhanced stemness and superior plasticity. In this study, the effect of cryopreservation on viability, stemness, and osteogenic differentiation capability of spontaneous CB-MSC spheroids were investigated. CB-MSCs were isolated from mouse femur and tibia. Spheroids were cryopreserved with various concentrations of dimethyl sulfoxide (DMSO). After thawing, the number of living and dead cells was measured. The expression levels of stem cell markers and osteogenic marker genes were analyzed. The cryopreserved and noncryopreserved spheroids were transplanted in mice with a beta-tricalcium phosphate as a scaffold to evaluate the *in vivo* bone-forming capability. The percentage of living cells was highest when 5% DMSO was used as a cryoprotectant, confirmed by the number of dead cells. The expression of stem cell marker genes and osteogenic differentiation capability were maintained after cryopreservation with 5% DMSO. The cryopreserved spontaneous CB-MSC spheroids showed remarkable new bone formation *in vivo*, identical to that of the noncryopreserved spheroids even without osteogenic induction. The cryopreserved spontaneous CB-MSC spheroids retained stemness and osteogenic differentiation capability and highlight the utility of spontaneous CB-MSC spheroids as ready-to-use tissue-engineered products for bone tissue engineering.

**Impact statement:**

The optimal dimethyl sulfoxide concentration for compact bone-derived mesenchymal stromal cells (CB-MSC) spheroid cryopreservation was determined. Cryopreservation did not affect the stemness and osteogenic capability of CB-MSC spheroids. Cryopreserved CB-MSC spheroids can be used for bone tissue engineering immediately after thawing without osteogenic induction.

## Introduction

Mesenchymal stromal cells (MSCs) are widely used in regenerative medicine, as they are considered relatively safe and pose no prominent ethical concerns.^1–3^ As a cell source for MSCs, bone marrow has been almost exclusively used until 2008.^4,5^ Then a growing number of novel MSC products derived from tissues other than bone marrow such as adipose tissue,^4,6^ perinatal tissue,^4^ and dental pulp^7^ have been applied clinically thereafter. However, recent studies have shown the advantage of compact bone as an alternative cell source for MSCs.^8^ Compact bone-derived mesenchymal stromal cells (CB-MSCs) harvested from small compact bone tips after enzymatic digestion possess superior stem cell properties and have enhanced osteogenic capabilities compared with bone marrow-derived MSCs.^9,10^ In particular, the spontaneously formed spheroids from CB-MSCs showed higher stem cell marker expression such as fucosyltransferase 4 (*Fut4* [*SSEA1*]), SRY (sex determining region Y)-box 2 (*Sox2*), *Nanog*, and *Casp3* (*SCA-1*) and showed significantly higher osteogenic marker expression after induction than monolayer-cultured cells, highlighting the usefulness of this cell source for bone tissue engineering.^11^

Although MSCs have been cultured as an adherent cell population and expanded in two-dimensional culture dishes, accumulating evidence has demonstrated the superiority of three-dimensional floating cell cultures, called spheroids or spheres.^12,13^ Spheroids have greater pluripotency, proliferative ability, and differentiation capability than monolayer cultured cells.^14,15^ Studies have shown the superior functionality of spheroid-forming cells such as enhanced anti-inflammatory^16^ and angiogenetic effects^17^ and post-transplantation survival rate.^15^ Accordingly, spheroids from MSCs will be used for the treatment of various diseases and are expected to replace existing monolayer cultured cell therapies.

We developed a novel method for efficient spheroid generation using a culture plate with specifically low adherence.^18^ This technique allows spontaneous spheroid formation from various cell sources including MSCs with superior stemness and differentiation capability without physical stimuli and specific growth factors such as epidermal growth factor and basic fibroblast growth factor (bFGF).^18^ Accordingly, this technique is easy and cost-effective, enabling a broader application of somatic stem cells.

One of the disadvantages of cell-based therapy is the difficulty of scheduling for cell processing, as living cells cannot be stored for long and should be used immediately after arrival. The scheduling of cell processing should be coordinated with the patients, doctors, and hospitals, making immediate schedule changes impractical. Cryopreservation of the cells or tissue-engineered products offers a fundamental solution to this problem as cells can be processed in advance and delivered upon request. However, it has been reported that cryobanked MSCs have reduced immunomodulatory and blood regulatory properties directly after thawing.^19,20^ In addition, proper cryopreservation protocols for tissue-engineered products are still poorly established. Although efficient methods for cryopreservation and storage of stem cells have been developed,^21,22^ knowledge about spheroid cryopreservation is limited.^23,24^

The aim of this study was to establish optimal conditions for the cryopreservation of spontaneous CB-MSC spheroids. To establish a standardized protocol for cryopreservation of spontaneous CB-MSC spheroids, we analyzed the stemness and differentiation capabilities of different cryopreservation conditions for spontaneous CB-MSC spheroids and compared them with noncryopreserved spheroids.

## Materials and Methods

### Animals

Experiments were performed using C57BL/6J mice (male, 3 weeks old) and C.B-17/IcrHsd-Prkdc^scid^ mice (SCID, male, 6 weeks old) (both from Japan SLC, Inc., Hamamatsu, Japan). Animals were housed at a specific pathogen-free environment in the animal experiment facility of Matsumoto Dental University High-Tech Center. The animals were kept under controlled environmental conditions at 22°C and 50% humidity with a 12 h light/dark cycle, with free access to food and water. All procedures and experiments in this study were performed in accordance with the guidelines laid down by the National Institutes of Health (NIH) in the United States, regarding the care and use of animals for experimental procedures. This study was approved by the Matsumoto Dental University Committee on Intramural Animal Use (Nos. 289 and 364).

### Isolation and culture of CB-MSCs

CB-MSCs were isolated and cultured according to previously published protocols.^10,11^ In brief, C57BL/6J mice were sacrificed with an overdose of pentobarbital. The femurs and tibiae were excised. Epiphyses were cut, and bone marrow was flushed out using a 27-gauge needle and syringe. Bone tissues were transferred into phosphate-buffered saline [PBS (-); FUJIFILM Wako Pure Chemical Corporation, Osaka, Japan], cut into 1–2 mm fragments with scissors, and incubated with 0.25% collagenase (FUJIFILM Wako Pure Chemical Corporation) for 45 min. The culture medium containing cells was collected into another centrifuge tube through a 40 μm cell strainer.

The cells were cultured in α-minimum essential medium (FUJIFILM Wako Pure Chemical Corporation) supplemented with 10% fetal bovine serum (FBS), 1% penicillin–streptomycin–amphotericin solution, and 10 ng/mL recombinant human bFGF (PeproTech, Rocky Hill, NJ, USA), which was used as a basic culture medium in this study. When the adherent cells (representing CB-MSCs) reached 80% confluence, the cells were trypsinized and resuspended in freeze-preserving medium (STEM-CELLBANKER; Takara Bio, Inc., Tokyo, Japan) until use.

### Spontaneous spheroid formation

Passage 2 cells were collected and seeded into a 55-mm spheroid-forming low adhesive culture dish (AS ONE, Osaka, Japan) at a density of 1.5 × 10^4^ cells/cm^2^ according to our previous report^8^. CB-MSCs spheroids formed after 24 h and used in the following study.

### Assessment of pluripotency markers

#### Alkaline phosphatase staining

Alkaline phosphatase (ALP) staining was performed using a kit (AP Staining Kit, No. AP100B-1; SBI, Palo Alto, CA, USA) according to the manufacture's instruction.^10^ Photomicrographs were obtained with an inverted system microscope (IX71; Olympus, Tokyo, Japan).

#### Immunofluorescence

Immunofluorescence was conducted according to the protocol in our previous publication.^10^ In brief, spheroids were collected and solidified by iPGell (Genostaff Co., Ltd., Tokyo, Japan) according to the manufacture's instruction. The samples were fixed with 4% paraformaldehyde in phosphate buffer (pH 7.4; FUJIFILM Wako Pure Chemical Corporation), dehydrated, and embedded in paraffin. Sections (8 μm in thickness) were cut.

After deparaffinization and rehydration, the sections were incubated with primary antibodies against pluripotency markers: Nanog (1:100; No. ab80892; Abcam), Sox2 (1:250; No. ab97959; Abcam), octamer-binding transcription factor 4 (Oct4; 1:250; No. ab19857; Abcam), and SSEA1 (1:100; No. ab16285; Abcam) overnight at 4°C. The sections were then washed in PBS three times. Secondary antibodies conjugated to Alexa Fluor (ab150121 and ab150079; Abcam) were added for 2 h at room temperature in the dark. Nuclei were counterstained with 4′,6-diamidino-2-phenylindole (DAPI) (ab104139; Abcam) for 30 min. The images were captured with a fluorescence microscope (KEYENCE BZ-X710; Keyence, Osaka, Japan).

### Optimization of cryoprotectant concentration for spontaneous CB-MSC spheroids

#### Cryopreservation

To investigate the effect of cryopreservation, we decided to optimize the condition for spheroid cryopreservation. As the final goal of this study was to develop optimal spheroid cryopreservation conditions for clinical usage, we chose dimethyl sulfoxide (DMSO) as a cryoprotectant, widely applied under clinical settings.^23^

Approximately 2.1 × 10^6^ CB-MSCs formed spontaneous CB-MSCs spheroids that were collected and divided into seven subgroups: 1% DMSO group (1% DMSO +99% FBS), 5% DMSO group (5% DMSO +95% FBS), 10% DMSO group (10% DMSO +90% FBS), 15% DMSO group (15% DMSO +85% FBS), 20% DMSO group (20% DMSO +80% FBS), stem-C group (STEM-CELLBANKER; used as a conventional cryoprotectant), fresh group (without cryopreservation and served as a control). The samples were transferred into cryogenic vials (Greiner Bio-One, Frichenhausen, Germany) and placed into a freezing vessel (Bicell, Nihon Freezer, Tokyo, Japan), and stored at −80°C for 24 h before transfer into liquid nitrogen.

#### Measurement of number of viable and dead cells

After 7 days of cryopreservation, the spheroids were quickly thawed in a 37°C water bath and immediately transferred into a 50 mL conical tube with 10 mL of basic culture medium. The spheroids were washed three times and transferred into 48-well plates. The number of viable and dead cells was measured at 3 h after thawing using the Viability/Cytotoxicity Multiplex Assay Kit (Dojindo, Kumamoto, Japan) based on [2-(2-methoxy-4-nitrophenyl)-3-(4-nitrophenyl)-5-(2,4-disulfophenyl)-2H-tetrazolium, monosodium salt] (WST-2) and lactate dehydrogenase (LDH) assays according to the manufacturer's instruction. The percentages of viable and dead cells were calculated relative to the viable cells in the noncryopreserved fresh group. The number of viable cells at 24 and 72 h was measured and the time course of cell growth was calculated as a percentage of initial number of cells.

#### Histological assessment of apoptotic cells and proliferating cells

Cryopreserved and noncryopreserved spheroids were solidified in iPGell and processed as described in Materials and Methods section. TdT-mediated dUTP Nick-End labeling (TUNEL) staining was used to detect apoptotic cells and the number of apoptotic cells with a kit (In Situ Cell Death Detection Kit, Fluorescein, Cat. No. 11684795910; Roche, Switzerland) according to the manufacturer's protocol. Immunofluorescence against Ki67 was used to detect proliferating cells (GB111141; Servicebio, Wuhan, China). The percentages of apoptotic cells were calculated from the average of three sections per group.

### Quantitative reverse transcription-polymerase chain reaction

Since the 5% DMSO group showed the highest cell survival rate and least number of apoptotic cells, this condition was used for the following experiment. RNA extraction and quantitative reverse transcription-polymerase chain reaction (qRT-PCR) were carried out as previously reported.^10^ In brief, total RNA was extracted using TRIzol (Invitrogen, Carlsbad, CA, USA). RNA samples were reverse transcribed into complementary DNA using oligo (dT) 12–18 primers (Life Technologies), deoxy-ribonucleoside triphosphate (dNTP) (Toyobo Co., Ltd., Osaka, Japan), and ReverTra Ace^®^ (Toyobo Co., Ltd.) according to the manufacturer's instructions. qRT-PCR was performed in a thermal cycler (Thermal Cycler Dice Real-Time System II TP-900; Takara Bio) using SYBR Premix Ex TaqII reagent (Takara Bio) according to the manufacturer's protocol.

Each qRT-PCR was done in triplicate, and the relative expression levels of genes were normalized against glyceraldehyde 3-phosphate dehydrogenase. Quantification was performed using the comparative Ct (2^-ΔΔCt^) method. Primer sets (Sigma-Aldrich Co.) used for the qRT-PCR experiment are listed in Table 1.

**Table 1.** Quantitative Reverse Transcription-Polymerase Chain Reaction Primer List

Primer	Direction	Sequence (5′-3′)
*Gapdh*	Forward	TGTGTCCGTCGTGGATCTGA
	Reverse	TTGCTGTTGAAGTCGCAGGAG
*Itgb1(CD29)*	Forward	CCATGCCAGGGACTGACAGA
	Reverse	GAGCTTGATTCCAATGGTCCAGA
*Cd44*	Forward	CAAGCCACTCTGGGATTGGTC
	Reverse	GGCAAGCAATGTCCTACCACAAC
*Eng (CD105)*	Forward	CTGCCAATGCTGTGCGTGAA
	Reverse	GCTGGAGTCGTAGGCCAAGT
*Casp3 (SCA-1)*	Forward	TTGCCTTTATAGCCCCTGCT
	Reverse	GTCATGAGCAGCAATCCACA
*Fut4 (SSEA1)*	Forward	GCAGGGCCCAAGATTAACTGAC
	Reverse	AAGCGCCTGGGCCTAAGAA
*Sox2*	Forward	GTTCTAGTGGTACGTTAGGCGCTTC
	Reverse	TCGCCCGGAGTCTAGCTCTAAATA
*Pou5f1 (Oct4)*	Forward	GTTGGAGAAGGTGGAACCAA
	Reverse	AGATGGTGGTCTGGCTGAAC
*Nanog*	Forward	CACCCACCCATGCTAGTCTT
	Reverse	ACCCTCAAACTCCTGGTCCT
*Klf4*	Forward	AACATGCCCGGACTTACAAA
	Reverse	TTCAAGGGAATCCTGGTCTTC
*Spp1 (Opn)*	Forward	TGCACCCAGATCCTATAGCC
	Reverse	CTCCATCGTCATCATCATCG
*Bglap2 (OCN)*	Forward	AAGCAGGAGGGCAATAAGGT
	Reverse	TGCCAGAGTTTGGCTTTAGG
*Col1a1*	Forward	GAGCGGAGAGTACTGGATCG
	Reverse	GCTTCTTTTCCTTGGGGTTC
*Ibsp (BSP)*	Forward	GAGACGGCGATAGTTCC
	Reverse	AGTGCCGCTAACTCAA
*Sp7 (Osterix)*	Forward	AGGCCTTTGCCAGTGCCTA
	Reverse	GCCAGATGGAAGCTGTGAAGA
*Dmp1*	Forward	AGTGAGTCATCAGAAGAAAGTCAAGC
	Reverse	CTATACTGGCCTCTGTCGTAGCC

### Induction and assessment of osteogenic differentiation

#### Induction of osteogenic differentiation

The spheroids from 5% DMSO (after freeze and thaw cycle) and fresh groups were transferred into a 48-well culture plate (Falcon^®^; Corning, NY, USA), with each group containing six samples. After the cell density reached 60% confluence, the culture media of three samples were changed to the osteogenic induction medium,^10,11^ and the other three samples were cultured continuously with the basic culture medium. The media were changed every 2 days. The markers for osteogenic differentiation were assessed after 7 days.

#### ALP staining

For the detection of ALP-positive cells after osteogenic induction, Alkaline Phosphatase Staining Kit (AK20; Cosmo Bio Co., Ltd., Tokyo, Japan) was used as instructed. In brief, the cells were washed with PBS and fixed with 10% formalin neutral buffer solution for 20 min. Then the cells were washed with purified water. The staining reagent was added to the cells and incubated for 20 min at 37°C. After removal of the staining reagent, the cells were washed with purified water and observed under a phase-contrast microscope (Olympus IX70; Olympus Optical Co., Ltd., Tokyo, Japan).

#### ALP activity assay

To analyze ALP activity, a WST-8 assay (Cell Counting Kit-8; Dojindo) and *p*-nitrophenyl phosphate (SIGMAFAST™ p-Nitrophenyl Phosphate Tablet; Sigma-Aldrich Co., LLC.) were used according to the manufacturer's instructions.^11^ Cell Counting Kit-8 was measured at 450 nm and *p*-nitrophenyl phosphate was measured at 405 nm using an iMark™ microplate absorbance reader (Bio-Rad Laboratories, Hercules, CA, USA). The ALP activity was calculated as a relative value of *p*-nitrophenyl phosphate to WST-8. Each experiment was carried out three times independently.

### *In vivo* transplantation experiment

#### Transplantation procedure

Six-week-old male SCID (C.B-17/IcrHsd-Prkdc^SCID^, *n* = 7) mice were used as recipients of heterotopic transplantation. In this study, beta-tricalcium phosphate (β-TCP) disks (2 mm in diameter and 2 mm in thickness) (OSferion; Olympus Terumo Biomaterials Corp., Tokyo, Japan) were used. Four groups were generated. (1) Noninduced fresh group; the fresh (noncryopreserved) spheroids were directly seeded onto the scaffold and transplanted without osteogenic induction. (2) Noninduced DMSO group; the cryopreserved spheroids were directly seeded onto the scaffold and transplanted without osteogenic induction. (3) Induced fresh group; the fresh (noncryopreserved) spheroids were seeded onto the scaffold and cultured in the osteogenic induction medium for 7 days before transplantation. (4) Induced DMSO group; the cryopreserved spheroids were seeded onto the scaffold and cultured in the osteogenic induction medium for 7 days before transplantation.

The samples were transplanted into the back of SCID mice as described previously.^10^ In brief, under general anesthesia with an intraperitoneal injection of pentobarbital sodium (65 mg/kg; Somnopentyl^®^; Kyoritsu Seiyaku Corp., Tsukuba, Japan), a subcutaneous incision was made at the middle of the dorsum. Four subcutaneous pockets were bluntly created at both sides of the caudal and dorsal regions. The scaffold with the CB-MSCs was transplanted into the pockets and the wound was sutured. As a control, β-TCP scaffolds without cells were also transplanted. The mice were sacrificed at 4 weeks after transplantation and the transplants were harvested.

#### Histological analysis of transplants

The samples were fixed in a 10% neutral-buffered formalin solution for 24 h, decalcified with 10% neutral buffered ethylene diamine tetraacetic acid (pH 7.1–7.5) for 14 days at room temperature. Then, the samples were embedded in paraffin, sectioned, and stained with hematoxylin and eosin (HE). Bone areas were analyzed using Image J software (National Institutes of Health, Bethesda, MD, USA). At least five sections were analyzed for each sample.

### Statistical analysis

Data were analyzed by statistical package for social science (SPSS) version 17.0. Kruskal–Wallis test was used for multiple comparisons. The Student's *t*-test was used for the analyses of qRT-PCR, ALP activity, and histomorphometric analyses to compare two groups. Statistical significance was set as *p* < 0.05.

## Results

### Characterization of spontaneous CB-MSC spheroid

**FIG. 1.**
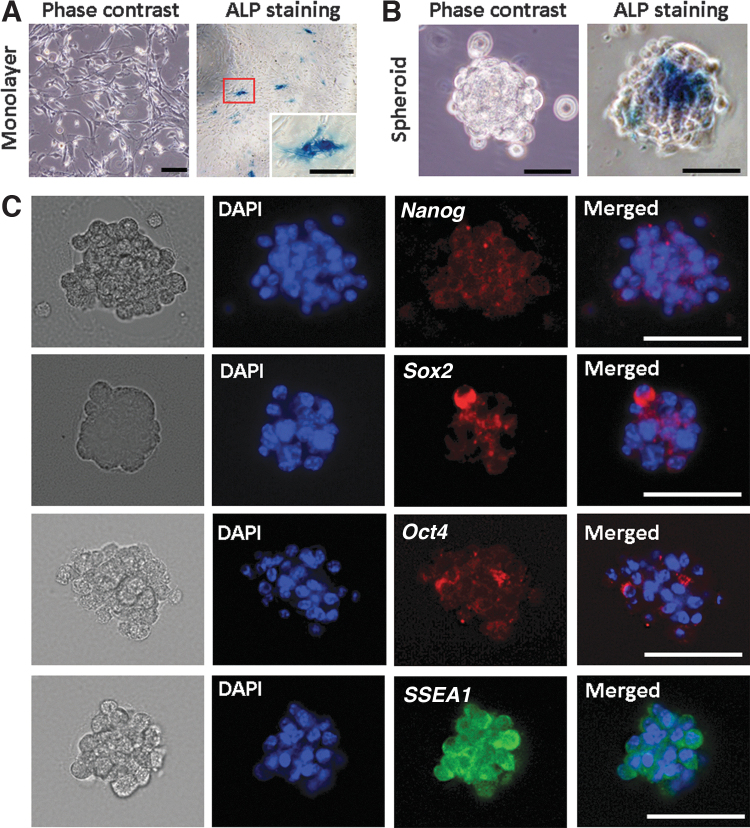
Characterization of the spontaneous CB-MSC spheroid. Phase-contrast photomicrographs showing ALP staining of monolayer cultured cells **(A)** and spheroid **(B)**. A high concentration of ALP-positive cells was observed in the middle of spontaneous CB-MSCs spheroid. Spontaneous CB-MSCs spheroids were positive for pluripotency markers: Nanog, Sox2, Oct4, and SSEA1 demonstrated **(C)**. Scale bar: 100 μm **(A)**; 50 μm **(B, C)**. ALP, alkaline phosphatase; CB-MSCs, compact bone-derived mesenchymal stromal cells.

CB-MSCs displayed typical fibroblast-like spindle shape morphology and were sparsely ALP positive in monolayer culture (Fig. 1A). After culture in the spheroid-forming plate for 24 h, the spheroid formation was observed and intense ALP expression was observed in the middle of spheroid-forming cells (Fig. 1B). To confirm the characteristics of spontaneous CB-MSCs spheroid further, the tissue sections from spheroids were immunostained with pluripotent stem cell markers. Many if not most of the spheroid-forming cells were positive for *Nanog*, *Sox2*, *Oct4*, and *SSEA1*, which confirmed the presence of abundant stem cells in CB-MSC spheroids (Fig. 1C).

### Optimization of cryoprotectant concentration for spontaneous CB-MSC spheroids

**FIG. 2.**
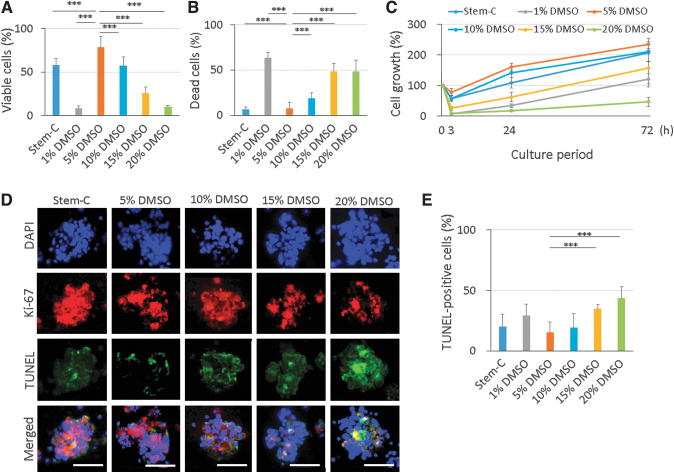
Effect of the cryoprotectant concentrations on cell viability after a freeze and thaw cycle. Different concentrations of DMSO (1%, 5%, 10%, 15%, and 20%) were assessed, with stem-C used as a conventional cryoprotectant for comparison. The percentages of viable cells were measured using the WST-8 assay **(A)**. The highest percentage of viable cells was observed in the 5% DMSO group. The percentages of dead cells were measured using the LDH assay **(B)**. The lowest percentages of dead cells were observed in the 5% DMSO group. Cell growth was measured at 3, 24, and 72 h after thawing **(C)**. The highest cell growth was observed in the 5% DMSO group. Immunofluorescence for Ki-67 and TUNEL staining was performed **(D)**. Scale bar: 50 μm. The percentage of TUNEL-positive cells was measured **(E)**. The lowest number of apoptotic cells was observed in the 5% DMSO group. Data are presented as mean ± SD. *n* = 5. ***p < 0.001. DMSO, dimethyl sulfoxide; LDH; SD, standard deviation; TUNEL; WST.

Cell viability was highest in the 5% DMSO group (78.3%), followed by the stem-C (57.8%) and the 10% DMSO (57.6%) groups (Fig. 2A). There were statistical differences between the 5% DMSO and other groups (*p* < 0.0001). This result was confirmed by the percentage of dead cells. The lowest cell death was observed in the 5% DMSO group (7.9%), followed by the stem-C group (6.5%) (Fig. 2B). The 1% DMSO group showed the highest percentage of dead cells. Cell growth was measured as a percentage of the initial number of cells (Fig. 2C). Number of cells declined 3 h after thawing in all groups and subsequently increased. The cell growth was fastest in the 5% DMSO group, followed by the 10% DMSO and stem-C groups. Cell growth was slowest in the 20% DMSO group. This tendency was maintained throughout the observation period.

To investigate the cytotoxicity of cryoprotectant further, the distribution of apoptotic and proliferating cells was evaluated using immunofluorescence for Ki67 and TUNEL staining. The Ki67-positive cells were observed in all groups but most abundant in the stem-C group followed by the 5% DMSO and the 10% DMSO groups. Fewer positive cells were observed in the 15% DMSO and the 20% DMSO groups. The proliferating cells did not show distinct localization and were observed throughout the whole area except for the central part of the 20% DMSO group, where no positive cells were found.

The results from the TUNEL staining showed fewer positive cells in the 5% DMSO group. The positive cells were abundant in the 20% DMSO group and intense staining was observed in the middle of the spheroids (Fig. 2D). The percentage of apoptotic cells was lowest in the 5% DMSO group (15.3%) and was significantly different from the 15% DMSO (34.9%; *p* < 0.001) and the 20% DMSO (43.5%; *p* < 0.001) groups (Fig. 2E).

Taken together, the 5% DMSO group showed the highest cell viability with the lowest percentage of dead and apoptotic cells and highest cell growth. Thus, we considered this set of conditions as optimal cryoprotectants for spontaneous CM-MSC spheroids among others.

### Effect of spontaneous CB-MSC spheroid cryopreservation on stemness

**FIG. 3.**
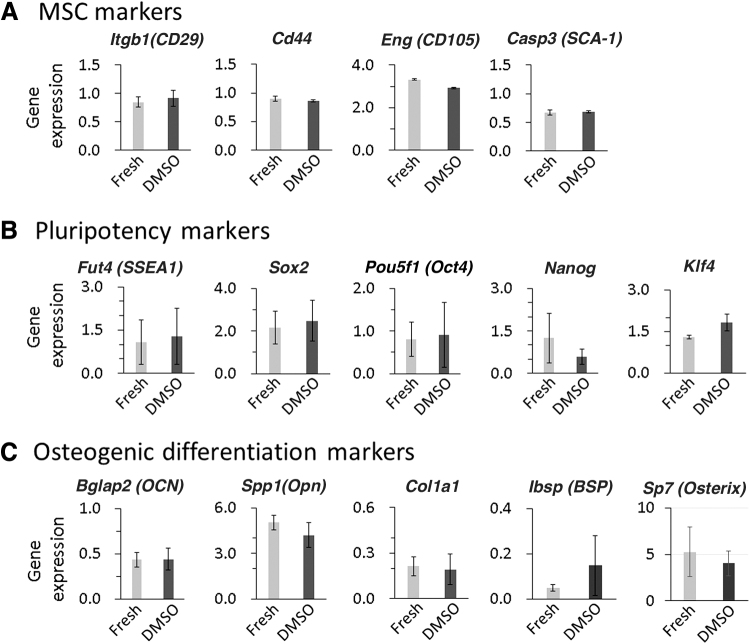
Effect of spheroid cryopreservation on stemness. The results from qRT-PCR for MSC markers **(A)**, pluripotency markers **(B)**, and osteogenic markers **(C)** are shown. There were no significant differences in expression of MSC markers (*CD29*, *Cd44*, *CD105*, and *Casp3 (SCA-1)*), pluripotency markers (*Fut4 (SSEA1)*, *Sox2*, *Oct4*, *Nanog*, and *Klf4*), and osteogenic differentiation markers (*OCN*, *OPN*, *Col1a1*, *BSP*, and *Osterix*) between the fresh and cryopreserved 5% DMSO groups. Data are expressed as the mean ± SEM, *n* = 3. MSC, mesenchymal stromal cells; qRT-PCR, quantitative reverse transcription-polymerase chain reaction; SEM, standard error of the mean.

Based on the mentioned experiments, 5% DMSO was best among the tested experimental conditions and used for the following experiments. The expression of stem cell markers of cryopreserved spheroids was analyzed and compared with those of the fresh group. The expression of MSC markers such as *CD29*, *CD44*, *CD105*, and *Casp3 (SCA-1)* was measured using qRT-PCR (Fig. 3A). No significant differences were observed between the 5% DMSO and fresh groups for all those markers. In terms of the pluripotency markers such as *Fut4 (SSEA1)*, *Sox2*, *Oct4*, *Nanog*, and Krüppel-like factor 4 (*Klf4*), there was no significant difference between the 5% DMSO and fresh groups (Fig. 3B).

The expression of osteogenic marker genes such as osteocalcin (*OCN*), osteopontin (*OPN*), collagen type I alpha 1 chain (*Col1a1*), bone sialoprotein (*BSP*), and *Osterix* was also analyzed. No significant differences were observed between the 5% DMSO and fresh groups (Fig. 3C). Overall, these results showed that the cryopreservation of spheroids under the optimal condition does not affect the stemness and the status of osteogenic differentiation.

### Effect of spontaneous CB-MSC spheroid cryopreservation on osteogenic differentiation capability

**FIG. 4.**
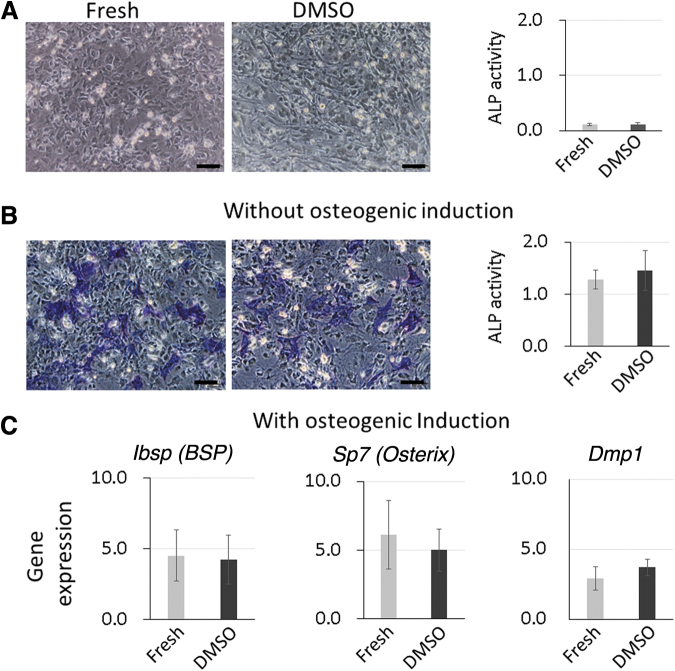
Osteogenic differentiation capability before and after cryopreservation. ALP staining and ALP activity for spontaneous CB-MSC spheroids without osteogenic induction **(A)**. No positive cells were observed. ALP activity was low and no significant difference was observed between the fresh and the cryopreserved spheroids (5% DMSO). After 7 days of osteogenic induction, the cells from both fresh and 5% DMSO groups showed ALP-positive cells. The staining was almost identical between the fresh and 5% DMSO groups. ALP activity was measured after osteogenic induction, with no significant differences between the fresh and the 5% DMSO groups **(B)**. BSP, Osterix, and DMP1 expression levels were measured after osteogenic induction, with no significant difference between the fresh and the 5% DMSO groups **(C)**. Scale bar: 100 μm. Data are expressed as the mean ± SD, *n* = 3.

To investigate the usefulness of cryopreserved spheroids further, the osteogenic capability was analyzed and compared with that of the fresh group. Without osteogenic induction, ALP staining showed no positive cells in both the cryopreserved 5% DMSO and fresh groups (Fig. 4A). Without osteogenic induction, the ALP activity of the cryopreserved 5% DMSO and fresh groups was identical and remained very low (Fig. 4A).

After 7 days of osteogenic induction, the cryopreserved group exhibited ALP-positive cells, identical to that of the fresh group (Fig. 4B). After osteogenic induction, the ALP activity was higher than those without osteogenic induction in both conditions (Fig. 4A, B) However, ALP activities in the cryopreserved 5% DMSO and fresh groups showed no significant difference (Fig. 4B).

In parallel with these results, there was no significant difference in the expression of BSP, Osterix, and dentin matrix protein 1 (DMP1) between the 5% DMSO- and fresh group-derived cells after osteogenic induction (Fig. 4C). Taken together, the cryopreservation did not affect the osteogenic differentiation capability.

### Effect of spontaneous CB-MSC spheroid cryopreservation on *in vivo* osteogenic capability

**FIG. 5.**
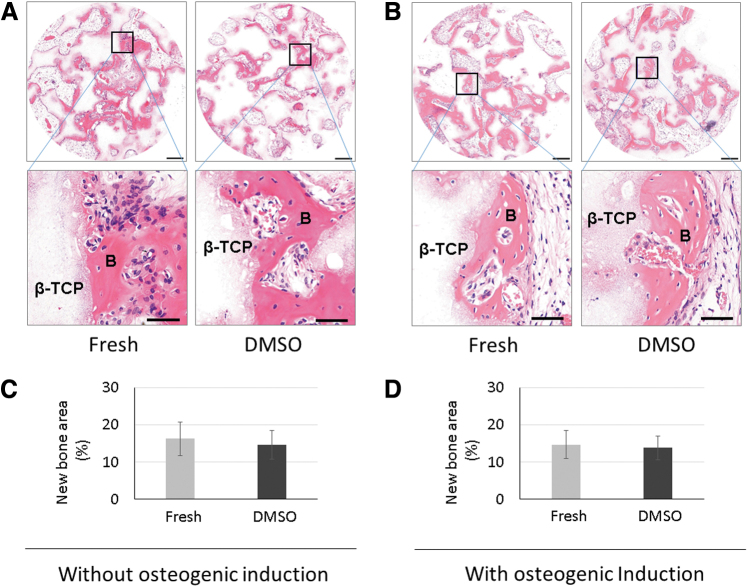
The *in vivo* bone-forming capability after cryopreservation. The bone formation was observed using HE staining of the sections from transplants. Fresh and cryopreserved (5% DMSO) spheroids were transplanted after cell seeding without osteogenic induction. Remarkably new bone formation was observed in the transplants from both the fresh and cryopreserved spheroids **(A)**. Next, the spheroids from the fresh and 5% DMSO groups were seeded onto a scaffold and transplanted after 7 days of osteogenic induction. Abundant new bone formation was observed in both the fresh and 5% DMSO groups **(B)**. Morphometric analyses showed no significant difference in the samples without osteogenic induction in the new bone area between the transplants from the fresh and cryopreserved groups **(C)**. The area of new bone was also identical between the fresh and cryopreserved groups when cultured in the osteogenic induction medium for 7 days **(D)**. Interestingly, the *in vivo* bone-forming capability was unaffected by osteogenic induction, with comparable new bone areas from the noninduced and induced groups. Data are expressed as the mean ± SD, *n* = 7. Scale bar: 200 μm (magnified images: 50 μm). HE, hematoxylin and eosin.

To evaluate the *in vivo* osteogenic capacity of spontaneous CB-MSC spheroids after cryopreservation, a transplantation experiment was performed. The cryopreserved (5% DMSO group) and noncryopreserved CB-MSC spheroids (fresh group) were seeded onto a β-TCP scaffold. The cells with scaffolds were transplanted directly or transplanted after 7 days of osteogenic induction to the back of mice subcutaneously. The samples were harvested after 4 weeks. The representative photomicrographs of HE staining are shown in Figure 5. The samples with the noninduced cells from both fresh and cryopreserved groups showed remarkable bone formation (Fig. 5A). The samples with induced cells also showed abundant new bone formation (Fig. 5B) and the level of new bone formation was similar between fresh and cryopreserved groups. These results were confirmed by morphometric analysis. Without osteogenic induction, the area of new bone formation in fresh and cryopreserved groups showed no significant difference (Fig. 5C). This tendency was similar to the samples with osteogenic induction and the new bone area showed no significant difference between fresh spheroid-derived cells and cryopreserved spheroid-derived cells (Fig. 5D). When the scaffolds were transplanted without cells, almost no bone formation was observed (Supplementary Fig. S1).

Interestingly, the *in vivo* bone-forming capability of spontaneous CB-MSC spheroids was not affected by the conduction of osteogenic induction and the new bone area was identical between the induced and noninduced samples in both fresh and cryopreserved groups (Fig. 5C, D). Overall, the osteogenic capability of spontaneous CB-MSC spheroids was not affected by the cryopreservation. Spontaneous CB-MSC spheroids do not require osteogenic induction to improve *in vivo* bone formation.

## Discussion

Cryopreservation processes cause cell death due to ice crystal formation, osmotic imbalance, and free radical production during the cooling and thawing steps.^25^ Cryoprotectants reduce ice crystal formation and act as solvents, diluting the intracellular electrolyte solution.^26,27^ Cryoprotectants are categorized into two groups, permeating and nonpermeating chemicals. DMSO is a permeating chemical and has been used commonly due to its excellent performance. Besides, due to the cytotoxicity and potential side effects,^28^ the development for DMSO-free cryoprotectants has been attempted. Although there are a number of promising candidates such as sucrose, glycerol, dextran, and trehalose, there is still no cryoprotectant that can replace DMSO under clinical circumstances.^28,29^ As we wanted to develop a protocol for the cryopreservation of spheroids for clinical application, we selected DMSO as a cryoprotectant.

Although the feasibility and potential drawbacks of MSC cryopreservation have been reported,^21,30^ the knowledge of spheroid cryopreservation is limited.^19,20,23,31^ In particular, the effect of cryopreservation on MSC spheroids is poorly understood.^23^ Despite their cryoprotective functions, most including DMSO are cytotoxic.^32,33^ As a result, cryoprotectant use has to be optimized based on its cryoprotective effect and cytotoxicity.^33,34^ Since spheroids consist of dozens to hundreds of cells, the optimal freezing protocol for a single cell suspension cannot be applied. The optimal conditions should be determined based on the size of spheroids and the type/status of cells.^35–37^ Use of lower concentrations of cryoprotectant causes insufficient permeation to the central part and may result in a lower survival rate. On the contrary, the use of higher concentrations of cryoprotectants may decrease the survival rate due to cytotoxicity.

Our results demonstrated that the 5% DMSO group had the highest cell survival, confirmed by the lowest percentage of dead and apoptotic cells. For the single-cell suspensions, the reported optimal concentrations of DMSO were between 5% and 10% for most of the cells under various freezing protocols.^36,38–40^ Interestingly, we found that the cell viability of the 10% DMSO group was significantly lower than that of the 5% DMSO group. Compared with the 5% DMSO group, the 1% DMSO group had decreased cell survival, which may reflect the insufficiency of cryoprotectant. DMSO concentrations higher than 15% also had lower cell survival and higher apoptotic cell ratio, demonstrating its cytotoxic effect at higher concentrations.

Based on these results, the relatively lower cell survival rate at 10% might be due to DMSO cytotoxicity. However, it is unclear why spheroids are more sensitive to DMSO than single-cell suspensions. Spontaneously formed spheroids from CB-MSCs used in this study express not only MSC markers but also pluripotency embryonic stem cell markers such as *Nanog*, *Sox2*, *Oct4*, and *Fut 4 (SSEA1)*, suggesting the superior stemness of spheroid-forming cells. The cell survival rate of human embryonic stem cells under the slow freezing process was low. It was reported that only 79% of the clumps were recovered after thawing, and only 16% developed after plating into undersized embryonic stem cell colonies with a high level of differentiation.^41^ The specific undifferentiated nature of spontaneous CB-MSC spheroids might cause increased sensitivity against DMSO.^42^

Another possibility is the effect of hypoxic environment, since the cells inside of spheroids are exposed to hypoxic environment. The accumulation of apoptotic cells in the central part of spheroids in the 20% DMSO group might support this hypothesis. However, the effect of hypoxic environment on DMSO cytotoxicity is unclear and further studies are required to clarify this mechanism.

The cell survival rate is an important index for the evaluation of the optimal freezing condition. However, it is also important that cryopreservation does not alter the function of cells. In this study, the expression levels of MSC markers and the pluripotency markers were compared in the spheroids with and without cryopreservation. Our results showed that the expression of stem cell markers was not affected by cryopreservation.

To investigate the effect of cryopreservation further, the osteogenic capability was also investigated. After osteogenic induction, the cryopreserved spheroid-derived cells exhibited comparable levels of ALP activity and Osterix expression with fresh spheroid-derived cells. Furthermore, cryopreserved spontaneous CB-MSC spheroids showed remarkable *in vivo* bone formation, which was identical to that of fresh CB-MSC spheroids.

Taken together, the cryopreservation of spontaneous CB-MSC spheroids under appropriate conditions did not affect their function. Interestingly, the amount of new bone formation of noninduced cells was identical to that of the fresh and cryopreserved induced CB-MSC spheroids. This suggests that functional skeletal stem cells in spontaneous CB-MSC spheroids were maintained after freeze and thaw cycles and that these cells can spontaneously differentiate into osteoblasts after transplantation.^43–45^ Although deciphering the detailed mechanism of this phenomenon was beyond the scope of this study, direct transplantation of cryopreserved spheroids and tissue-engineered products after thawing is cost-efficient and affords practical clinical application of tissue-engineered bone made with spontaneous CB-MSC spheroids.

## Conclusion

Our findings showed that the cryopreservation of spontaneous CB-MSC spheroids was feasible without damaging the stemness and function. Cryopreserved spheroids can be directly transplanted without osteogenic induction, enabling cost-efficient options for practical clinical applications for bone tissue engineering.

## Supplementary Material

Supplemental data
