# Supplementary figures and images for: Cryopreserved Spontaneous Spheroids from Compact Bone-Derived Mesenchymal Stromal Cells for Bone Tissue Engineering
Source: Tissue Eng Part C Methods. 2021 Apr 19;27(4):253–63. doi: 10.1089/ten.tec.2021.0001 (PMC8064946; doi:10.1089/ten.tec.2021.0001)

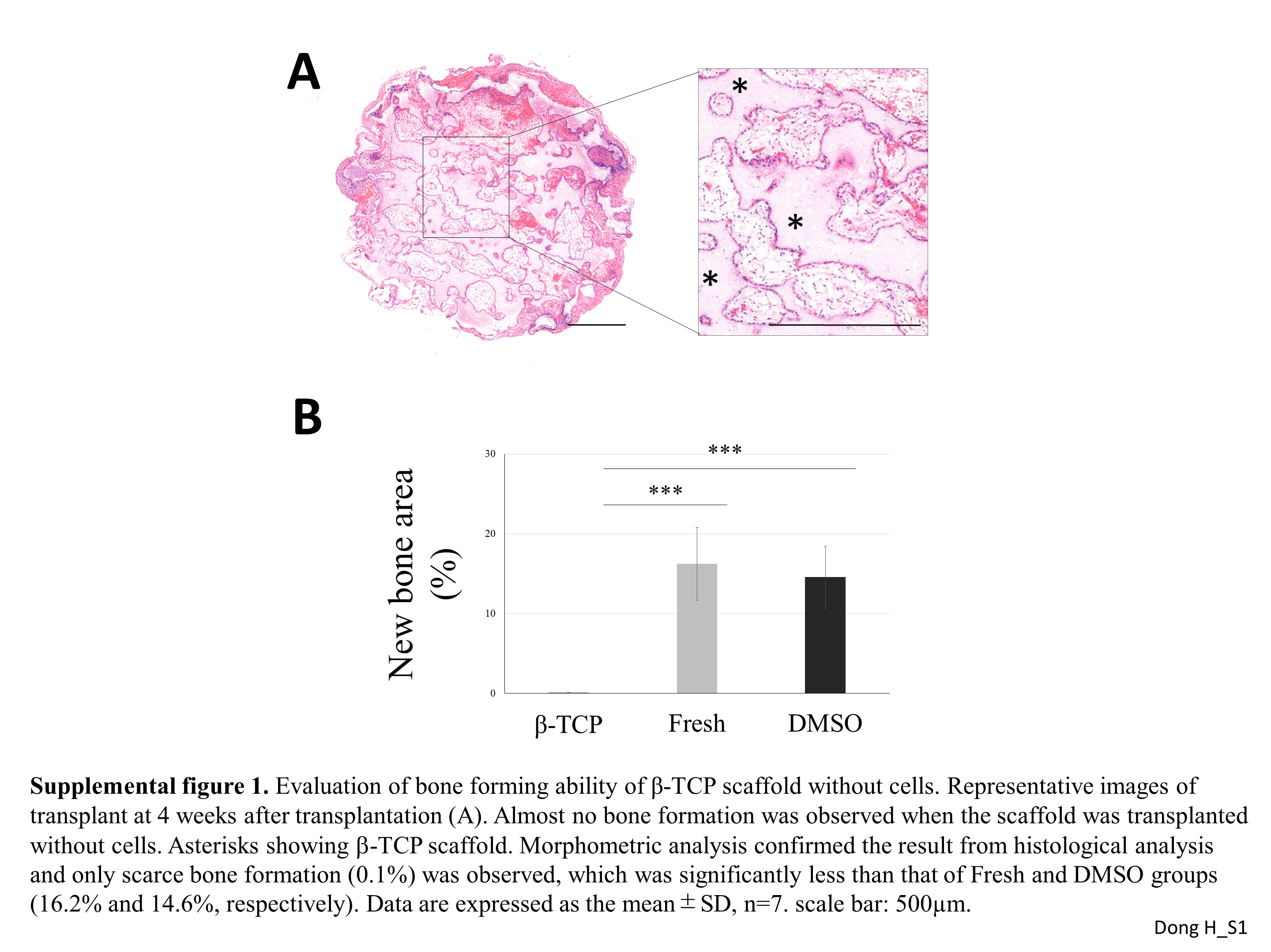

Supplement: Supplemental data [file Supp_FigS1.tif]
